# Association of Lower Antispike Antibody Levels with Mortality in ICU Patients with COVID-19 Disease

**DOI:** 10.1155/2023/4174241

**Published:** 2023-01-31

**Authors:** Sangeeta Yelle, Rahul Amte, Vishwanath Gella, Sasikala Mitnala, Deepika Gujjarlapudi, Mohammed Ismail, Ledo Thankachan, Sandhyarani Adla, Fatima Unnisa, Sivakumar Reddy, Duvvur Nageshwar Reddy

**Affiliations:** ^1^Critical Care Medicine, Asian Institute of Gastroenterology, Hyderabad, India; ^2^Pulmonology, Asian Institute of Gastroenterology, Hyderabad, India; ^3^Research Department, Asian Institute of Gastroenterology, Hyderabad, India; ^4^Biochemistry, Asian Institute of Gastroenterology, Hyderabad, India; ^5^Asian Institute of Gastroenterology, Hyderabad, India

## Abstract

**Background:**

Though vaccines have been reported as highly efficacious in preventing severe COVID-19 disease, there is emerging data of severe infections, albeit a small number, in vaccinated individuals. We have conducted a retrospective observational study to assess the clinical characteristics, immunological response, and disease outcomes among the vaccinated and unvaccinated patients admitted to the ICU with severe COVID-19 disease.

**Methods:**

*Study Design and Participants*. We conducted a retrospective observational study in COVID ICU of a tertiary care hospital. Data were collected from the month of 1 April 2021 to 31 November 2021. All adult patients admitted to the ICU having severe COVID-19 disease were included in the study. Data were collected from the medical records database which included demographics, a clinical course in the ICU, laboratory and radiological parameters, and disease outcomes. In a subset of patients, cell-mediated immunity and S1S2-neutralising antibody assessment was done.

**Results:**

A total of 419 patients with severe COVID-19 were included in the study. Of the 419 patients, 90 (21.5%) were vaccinated, and 329 (78.5%) were unvaccinated. There was a significantly higher mortality in unvaccinated severe COVID 19 patients as compared to vaccinated severe COVID patients (46.2% vs 34.4%; *P* < 0.0455). The neutralizing antibody titre was significantly higher in survivors as compared to nonsurvivors (2139.8, SE ± 713.3 vs 471, SE ± 154.4); *P* < 0.026.

**Conclusion:**

Our study suggests the association of lower neutralizing antibody levels with mortality in ICU patients admitted with COVID-19 breakthrough infections.

## 1. Introduction

Vaccines have been designed and rolled out to protect against severe COVID-19 disease and to reduce mortality. India initiated its vaccination program in the month of January, 2021. Oxford Astrazeneca Covidshield vaccine manufactured by Serum Institute of India and Covaxin (BBV152) developed by Bharat Biotech in collaboration with the Indian Council of Medical Research were the two vaccines initially available for mass vaccination. Covaxin is a whole inactivated virus vaccine. In a phase 3 trial, BBV152 was found to have overall efficacy of 77.8% (95% CI, 65.2%–86.4%) in preventing symptomatic disease [1]. As of 31 January 2020, Covaxin has been granted emergency use approval in 13 countries. It is recommended for individuals more than 6 years of age given as two doses 28 days apart. Covishield is a recombinant replication-deficient chimpanzee adenovirus vector vaccine containing genetic material of spike protein. It has an efficacy of 72% against symptomatic SARS-CoV-2 infection and is administered as 2 doses, 12 to 16 weeks apart in individuals more than 12 years of age [2]. For both Covishield and Covaxin, booster dose is recommended after 4 to 6 months.

Another vaccine approved in India is Sputnik V produced by Gamaleya Institute in Russia. It is a viral vector-based vaccine taken in 2 doses 21 days apart. Sputnik V was reported to have higher efficacy in phase 3 trial approaching that of mRNA vaccines [3]. Pfizer/biotech and Moderna were the foremost vaccines introduced with a reported vaccine efficacy of 95% against symptomatic disease [4]. They are mRNA vaccines, but usage in India seemed difficult because of the storage temperature required (must be stored at freezer-level temperature).

In spite of successful vaccination programs covering the population in general and at-risk individuals in particular, breakthrough infections causing severe disease and ICU admission have been reported [5–8]. Efficacy of the vaccines needs to be continuously monitored for efficient implementation of the vaccination program to minimize the severity of the disease and mortality [9]. Clinical data emanating from the real world based on the vaccination status of patients with severe disease admitted to ICU is sparse and less reported in the literature. We therefore conducted a retrospective, observational study to assess the clinical outcomes of ICU patients based on the vaccination status.

## 2. Patients and Methods

This is a single-centre retrospective, observational study conducted in the medical ICU of a tertiary care hospital in south India. Data were collected from RT-PCR-positive patients (>18 years of age) admitted to ICU with severe COVID-19 disease (*n* = 419) between April 2021 to November 2021, which included both vaccinated and unvaccinated patients. Exclusion criteria were those with unknown vaccination status, pregnant women, and patients or relatives who declined to consent to participate in the study.

All the patients received treatment as per standard protocols. Data were collected from the medical records database which included demographic characteristics, comorbidities, type of vaccine, inflammatory (D-dimer, ferritin, CRP, IL6, and LDH), hematological and biochemical parameters clinical course in the ICU, and disease outcome. The study was approved by the Institutional Ethics Committee of AIG Hospitals, Hyderabad, India.

In a subset of individuals, the immunological assessment was performed by evaluating neutralizing (antispike or S1S2) antibodies and enumeration of T and B cell responses. S1S2 IgG antibodies were enumerated on automated Diasorin Liaison XL by Chemiluminescence Immunoassay (CLIA) [10, 11]. The detection limit is ≥3.8 AU/ML, and the samples with ≥15 AU/ml were considered positive for neutralizing antibodies. For evaluating immune responses, peripheral mononuclear cells (PBMCs) were isolated using Hisep (Himedia) employing standard protocol. PBMCs were characterized phenotypically by flow cytometry for T and B lymphocytes [12]. CD3 (APC-H7), CD4 (Percp Cy 5.5), and CD8 (FITC) for T lymphocyte and CD20 (PE) for B lymphocyte were characterized [13]. Appropriate isotype-matched, nonreactive fluorochrome-conjugated antibodies were employed as controls. Analysis of cell populations was performed by means of direct immunofluorescence with a FACS Lyrics flow cytometry (BD), and data were calculated.

### 2.1. Definitions

A breakthrough infection was defined as the detection of SARS-CoV-2 on RT-PCR assay performed at hospital admission in vaccinated patients. According to CDC, people are considered fully vaccinated for COVID-19 ≥ 2 weeks after they have received the second dose in a 2-dose series (ChadOxnCoV or Covaxin), and unvaccinated people refer to individuals that have not completed a vaccination series or received a single-dose of concerned vaccine. The disease severity was defined as per the current Indian Council Medical Research (ICMR) guidelines (Clinical Guidance for Management of Adult COVID-19 Patients (icmr.gov.in)) [14]. Severe disease at admission was defined as patients with SpO2 < 90% or requirement of ICU care. Mortality parameters include in-hospital as well as patients discharged against medical advice with poor outcome.

### 2.2. Statistical Analysis

The baseline characteristic of the two groups was presented as mean and standard error (SE) for continuous variables. The categorical variables were presented as % (percentage) of frequency distribution. Student's *t*-test, chi-square test, and Fisher's exact test were used for comparison between two groups. Univariate and multivariate logistic regression analysis was applied to see the effect of risk factors on survival status. The risk factors in multiple logistic regression analysis were entered by enter method. The receiver operating characteristics curve (ROC) analysis was applied to see the effect of immunological factors on survival status in subgroup analysis. The statistical package for social sciences (SPSS 21st version) and Med Cal C were used for the analysis. A *P* value < 0.05 was considered statistically significant.

### 2.3. Outcomes

The primary outcome of the study was to compare 30-day mortality in vaccinated with unvaccinated severe COVID-19 disease patients in the ICU setting. Secondary outcomes included requirement of mechanical ventilation, vasopressor requirement, need for renal replacement therapy, and immunological assessment.

## 3. Results

### 3.1. Demographics

A total of 419 patients were admitted to the ICU with severe COVID-19 disease during the study period. Of these, 90 (21.5%) patients were vaccinated, and 329 (78.5%) patients were unvaccinated. The baseline characteristics of the patients are depicted in Table 1. A significant difference (*P*=0.0001) in the mean age was noted between the vaccinated and unvaccinated groups (61.9 ± 12.18 Vs. 54.4 ± 14.09 years) (Table 1). Both the groups were predominantly comprised of males with no significant difference between the vaccinated and unvaccinated groups (66.7% Vs. 70.8%; *P*=0.45). Majority of the patients had diabetes and hypertension in both the groups (Table 1). However, there was no significant difference in the number of individuals with diabetes mellitus in the vaccinated and unvaccinated groups (60% Vs. 57.8%; *P* < 0.703). Likewise, there was no significant difference in hypertension between the vaccinated and the unvaccinated groups (65.6% vs 57.8%; *P* < 0.180). Whole genomic sequencing of the SARS-CoV-2 virus identified that the strains were of the delta variant.

### 3.2. Clinical Outcomes in ICU Patients

**Figure 1:**
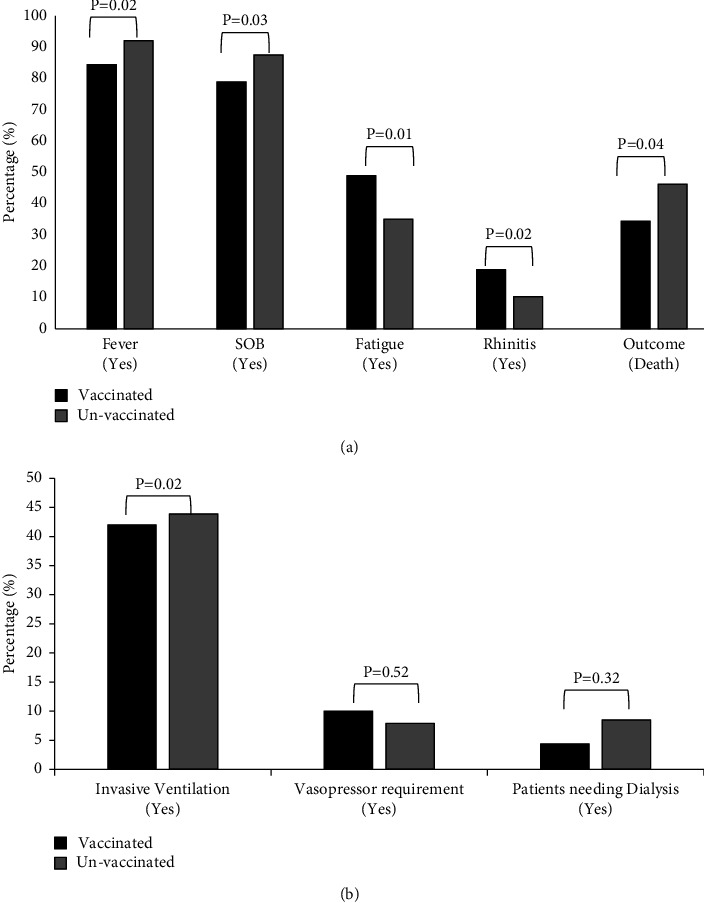
(a and b): depicting the comparison of clinical symptoms and organ support requirement between the vaccinated and the unvaccinated group by student *t*' test. SOB: shortness of breath.

There was significantly (*P* < 0.04) higher mortality in unvaccinated patients (152/329; 46.2%) as compared to vaccinated (31/90; 34.4%) patients with severe COVID-19. The vaccinated individuals had received either ChadOxnCoV (58.17%) or Covaxin (38.89%). There was no significant difference in mortality (*P* < 0.374) between the ChadOxnCoV (25.64%) or Covaxin (35.71%) groups. There was no significant difference in invasive ventilation requirement, vasopressor requirement, and the renal replacement therapy requirement between the vaccinated and unvaccinated groups as shown in Figure 1(b). The hospital length of stay was higher in vaccinated individuals as compared to unvaccinated individuals (17.3 vs 13.6, *P* < 0.001). As shown in Figure 1(a), a significantly higher proportion of individuals in the unvaccinated group had fever, shortness of breath (SOB), fatigue, and rhinitis as compared to the vaccinated patients with severe COVID-19 admitted to ICU.

### 3.3. Inflammatory Markers

Inflammatory markers such as D-dimer, ferritin, lactate dehydrogenase (LDH), and C reactive protein (CRP) were measured in these patients on admission to ICU. There was no significant difference in the D-dimer (1055 vs 1190.4, *P* < 0.485) and ferritin (900.3 vs 924.9, *P* < 0.847) levels among the vaccinated and unvaccinated groups (Table 2). LDH (902.1 vs 1024.7, *P* < 0.044) was found to be higher in the unvaccinated as compared to vaccinated groups. But CRP (116.9 vs 82, *P* < 0.00) was found to be higher in vaccinated group as compared to unvaccinated group.

**Figure 2:**
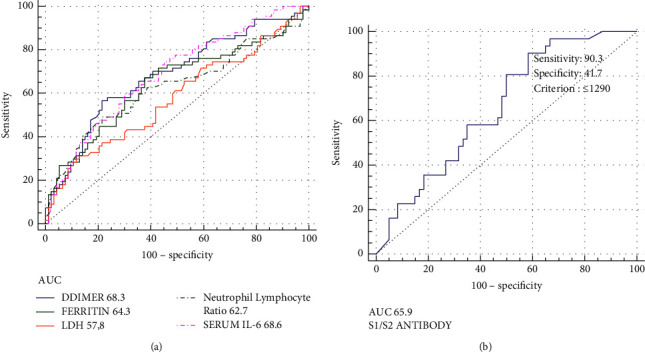
(a): ROC (receiver operator characteristic) curve for D-dimer, Ferritin, LDH, IL-6 and NeutrophilLymphotye Ratio as a predictor of mortality. (b): ROC Curve for S1S2 Neutralizing Antibodies as a predictor of mortality. Sensitivity of S1S2 antibody is higher (AUC: 65.9%) as compared to inflammatory markers. LDH = lactatedehydrogenase, IL6 = interleukin 6.

However, when comparison was made between nonsurvivors and survivors, these markers that are D-dimer (1522.5, SE ± 166.39 vs 883.1, SE ± 88.29, *P* < 0.000), ferritin (1180.4, SE ± 108.71 vs 719.5, SE ± 47.49, *P* < 0.000), LDH (1117.4, SE ± 47.63 vs 902.8, SE ± 29.29, *P* < 0.000), IL6 (135.5, SE ± 16.82 vs 65.3, SE ± 9.28, *P* < 0.000), and neutrophil (84.1%, SE ± 0.77 vs 80.5% ± 0.69, *P* < 0.000) were significantly higher in nonsurvivors (Figure 2(a) and Table 3).

The antispike antibody was available for 91 patients of whom 60 were survivors and 31 were nonsurvivors. The antibody titre was significantly higher in survivors (2139.8 ± 713.3) as compared to nonsurvivors (471.0 ± 154.4; *P* < 0.026). Univariate logistic regression analysis revealed the association of low S1/S2 antibody titre with increased risk of mortality (OR = 5.8, *P* < 0.008).

**Figure 3:**
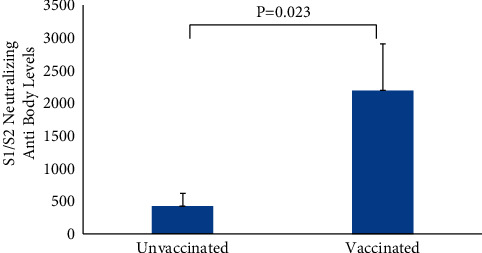
Comparison of S1S2 antibody titres between unvaccinated and vaccinated individuals by Student *t*' test.

The receiver operating curve (ROC) (Figure 2(b)) analysis showed the area under the curve (AUC) for S1S2 neutralizing antibodies to be 65.9% (0.54–0.77, *P* < 0.005). The sensitivity was 90.3%, and specificity was 41.7% for S1-/S2-neutralizing antibody titre. Also, among the 91 patients in whom antispike antibody levels were measured, 59 were vaccinated, and 32 were unvaccinated. The antibody titre was significantly higher in vaccinated (2182, SE ± 721.9) as compared to unvaccinated (445.01, SE ± 186.3, *P* < 0.023) (Figure 3).

However, there were 5 vaccinated patients who died despite having high neutralizing antibody levels, ranging from 658 AU/ML to 4225 AU/ML (details in Table 4).

There was no significant difference in the CD4 (3055 ± 431 Vs. 3705.0 ± 500.0; *P* < 0.327) and CD8 (1476.0 ± 226.0 Vs. 1470.0 ± 225.0, *P* < 0.0363) lymphocytes between the vaccinated and unvaccinated patients. However, a significant difference in the percentage of B cells between the vaccinated and the unvaccinated groups was noted (33.5 ± 3.35 Vs. 24.5 ± 2.76; *P* < 0.046).

## 4. Discussion

Global initiation of the vaccination programs has reduced the incidence of symptomatic disease, hospitalization, and death due to COVID-19 disease. Data pertaining to the clinical characteristics and outcomes in individuals developing severe disease as a result of breakthrough infection is limited. Our study describes the clinical and immunological parameters in patients admitted to ICU with severe COVID-19 after vaccination with either ChadOxnCoV-19 or Covaxin.

Higher mean age of the vaccinated group as compared to the unvaccinated group is likely due to the prioritization of elderly in the initial phases of the vaccination schedule by the Government of India. However, it is interesting to note that the number of vaccinated individuals hospitalized with severe disease is relatively lesser, and mortality is also lesser compared to the unvaccinated group in spite of the higher age during the study period pointing to the critical protective role of the vaccines against severe disease. The incidence of comorbidities was similar in the vaccinated and unvaccinated groups, but mortality was lower in vaccinated group. We found no significant difference in the need for mechanical ventilation, vasopressor, and RRT between the vaccinated and unvaccinated groups which may be attributable to comorbidities contributing to severe disease and organ dysfunction. It is intriguing to note that hospital length of stay was higher in vaccinated which is probably due to the reason that individuals in vaccinated group were elderly. However, these results have to be interpreted in light of data emanating from a single center.

In our cohort of unvaccinated patients admitted to ICU, the mortality was 46.2%. This is in agreement with the reported literature by Xie and Raquel et al. where mortality rates were between 38% and 64% in admitted ICU patients who were unvaccinated [15, 16]. However, interestingly, vaccination resulted in a significant decrease in mortality (34.4%, *P* < 0.045). We found no significant difference in mortality among the vaccinated ICU patients with regard to the two predominant vaccines being used in India. This indicates all vaccines protect against mortality even after individuals have developed severe diseases [17].

Immunogenicity studies have shown that antispike antibody levels whether elicited by vaccination or natural infection are a correlate of vaccine protection against COVID-19 disease [18]. The incidence of breakthrough infections is found to increase with waning immunity in older adults and clinical high-risk groups [19, 20] and with vaccine evasion by variant strains [21]. In a study by Gilbert et al. [22] evaluating SARS CoV 2 antibody markers as correlates of protection after mRNA-1273 vaccination against COVID-19, it was concluded that binding and neutralizing antibody markers strongly inversely correlated with COVID-19 risk and directly correlated with vaccine efficacy. Our study corroborates with this study that low S1/S2 antibody titre was associated with an increased risk of mortality.

Mortality was also seen in 5vaccinated patients who had high S1S2 antibody titres (Table 4). Mortality in these was attributed to factors such as presence of multiple comorbidities, secondary infections, or receipt of high dose of steroids. In a study conducted in US by CDC, 1,228,664 individuals who had completed primary vaccination were studied [23]. In these individuals, 189 (0.015%) had severe COVID-19 outcomes, and 36 (0.0033%) individuals died. They also noted that all individuals with severe outcomes were ≥ 65 years age, were immunosuppressed, or had at least one comorbidity.

Our study has limitations because it is a single center retrospective study. A prospective study with serial antibody measurement for evaluating the vaccine effectiveness and waning immunity may offer better insight. Another limitation is that the number of patients in unvaccinated group is higher. This is because in the initial phase, we saw the surge of delta virus affected cases who were largely unvaccinated cases. After the decline in the surge, we saw mixed vaccinated and unvaccinated cases in the ICU that is from mid-August onwards, that is, when we started measuring the antispike antibody levels as a marker of vaccine protection.

## 5. Conclusion

Our study lends support to the finding that even in ICU setting, vaccination is associated with lower mortality. Hence, our findings support the recommendation that antispike antibody levels be monitored for waning vaccine immunity. Future studies are needed to define an objective level of antispike antibody titres at which booster dose may be recommended especially in elderly individuals with comorbidities.

## Figures and Tables

**Table 1:** Comparison of demographic parameters and clinical outcome between vaccinated and unvaccinated by using *t* test and chi square test.

	Vaccinated (*n* = 90)	Unvaccinated (*n* = 329)	*P*-value
Age (years)	61.9 ± 1.28	54.4 ± 0.78	0.001^∗^
Body mass index	27.3 ± 2.68	25.7 ± 0.23	0.005^∗^
Gender (male)	60 (66.7%)	233 (70.8%)	0.453
Diabetes mellitus	54 (60%)	190 (57.8%)	0.704
Hypertension	59 (65.6%)	190 (57.8%)	0.18
Coronary artery disease	13 (14.4%)	56 (17%)	0.555
Thyroid	11 (12.2%)	43 (13.1%)	0.818
Rheumatological disease	0 (0%)	4 (1.2%)	0.298
Chronic liver disease	0 (0%)	9 (2.7%)	0.114
Bronchial asthma	5 (5.6%)	15 (4.6%)	0.697
Chronic obstructive pulmonary disease	2 (2.2%)	3 (0.9%)	0.313
Chronic kidney disease	10 (11.1%)	66 (20.1%)	0.05
Previous stroke	2 (2.2%)	1 (0.3%)	0.057
Outcome			
Death	31 (34.4%)	152 (46.2%)	0.046^∗^
Alive	59 (65.6%)	177 (53.9%)	
Hospital length of stay	17.3 ± 1.25	13.6 ± 0.47	0.001^∗^

Values are presented as Mean ± Standard Error and (%) as required. ^∗^*P* value < 0.05 is statistically significant.

**Table 2:** Comparison of inflammatory and laboratory parameters between vaccinated and unvaccinated using *t* test.

Parameter	Vaccinated		Unvaccinated		*P* value
	*N*	Mean ± SE	*N*	Mean ± SE	
D-dimer (ng/mL)	86	1055.0 ± 162.53	316	1190.4 ± 104.60	0.485
Ferritin (ng/mL)	81	900.3 ± 109.95	308	924.9 ± 63.90	0.847
Serum IL-6 levels (pg/mL)	66	84.8 ± 15.64	208	97.6 ± 10.89	0.504
Procalcitonin (ng/mL)	77	2.4 ± 1.11	257	1.4 ± 0.33	0.235
Lactate dehydrogenase (U/L)	77	902.1 ± 51.20	282	1024.7 ± 31.64	0.044^∗^
C-reactive protein (mg/L)	87	116.9 ± 9.43	295	82.0 ± 4.20	0.000^∗^
Hemoglobin (gm/dL)	89	11.9 ± 0.22	324	12.7 ± 0.11	0.003^∗^
Total leucocyte count (cells/mm^3^)	89	10041.8 ± 519.54	324	11396.0 ± 711.26	0.125
Absolute eosinophil count (cells/mm^3^)	86	90.1 ± 11.95	323	90.7 ± 6.17	0.963
Absolute monocyte count (cells/mm^3^)	86	455.4 ± 37.13	323	477.5 ± 22.30	0.611
Absolute neutrophil count (cells/mm^3^)	86	8592.6 ± 520.02	323	9013.0 ± 313.89	0.490
Absolute lymphocyte count (cells/mm^3^)	86	974.7 ± 49.69	323	975.0 ± 29.04	0.996
Neutrophil lymphocyte ratio	76	11.8 ± 1.15	184	13.1 ± 0.87	0.350
Platelet count (10^3^/mm^3^)	89	2.3 ± 0.09	325	187.0 ± 184.61	0.318
Blood urea (mg/dL)	87	47.2 ± 3.11	322	47.8 ± 1.68	0.863
Serum creatinine (mg/dL)	89	1.2 ± 0.13	323	1.4 ± 0.22	0.382
Sodium (mEq/L)	88	134.5 ± 0.56	322	135.7 ± 0.31	0.071
Potassium (mEq/L)	88	4.9 ± 0.51	322	4.3 ± 0.03	0.048
Total bilirubin (mg/dL)	87	0.8 ± 0.05	316	1.4 ± 0.34	0.061

*n* = number of persons. Values are expressed as mean ± Standard Error. ^∗^*P* value < 0.05 is statistically significant. ng/mL = nanogram per millilitre. pg/mL = picogram per millilitre. U/L = unit per litre. Cells/mm^3^ = cells per cubic millimetre. mg/L = milligram per litre. gm/dL = gram per decilitre. mEq/L = milliequivalents per litre.

**Table 3:** Comparison of inflammatory markers between survivors and non survivors by cross sectional univariate analysis.

S. no			Death		Alive		Odds ratio	*P* value	95% confidence interval	
			*n*	%	*N*	%			Lower	Upper

1	D-dimer	High risk	51	29.1	38	16.7	2.04	0.003^∗^	1.26	3.21
		Low risk	124	70.9	189	83.3				

2	Lactate dehydrogenase	High risk	76	47.5	68	34.2	2.12	0.001^∗^	1.13	2.67
		Low risk	84	52.5	131	65.8				

3	Ferritin	High risk	72	42.6	57	25.9	2.12	0.006^∗^	1.38	3.25
		Low risk	97	57.4	163	74.1				

4	C-reactive protein	High risk	65	40.6	77	34.7	1.28	0.236	0.85	1.95
		Low risk	95	59.4	145	65.3				

5	Neutrophils count	High risk	65	36.1	41	17.7	2.63	0.001^∗^	1.67	4.14
		Low risk	115	63.9	191	82.3				

6	Lymphocytes	High risk	58	32.2	113	48.7	0.51	0.001^∗^	0.33	0.75
		Low risk	122	67.8	119	51.3				

^∗^ *P* value < 0.05 is statistically significant. *N* = number of patients alive. *n* = number of patients dead.

**Table 4:** Characteristics of non-surviving patients with high antibody titres.

	Age (y)	Sex	Risk factors	Anti-spike antibody	D-dimer	Ferritin
1	50	M	High dose steroids	1120	4486	957.5
2	74	M	HTN, high dose steroids	658	555	69.5
3	79	M	HTN, DM, CKD, carcinoma prostate	1810	296	231.3
4	72	M	HTN, DM, morbid obesity	4225	5641	1316.4
5	64	F	HTN, DM	2070	2803	1252

HTN: hypertension, DM: diabetes mellitus, CKD: chronic kidney disease, *y*: years, M = male, F = female.

## Data Availability

The data used to support the findings of this study are included within the article.
